# Mitigating effects of vaccination on influenza outbreaks given constraints in stockpile size and daily administration capacity

**DOI:** 10.1186/1471-2334-11-207

**Published:** 2011-08-01

**Authors:** Maytee Cruz-Aponte, Erin C McKiernan, Marco A Herrera-Valdez

**Affiliations:** 1 Mathematical, Computational, and Modeling Sciences Center, Arizona State University, Tempe, AZ, USA; 2 School of Human Evolution and Social Change, Arizona State University, Tempe, AZ, USA; 3 School of Life Sciences, Arizona State University, Tempe, AZ, USA; 4 Institute of Interdisciplinary Research, University of Puerto Rico, Cayey, USA

## Abstract

**Background:**

Influenza viruses are a major cause of morbidity and mortality worldwide. Vaccination remains a powerful tool for preventing or mitigating influenza outbreaks. Yet, vaccine supplies and daily administration capacities are limited, even in developed countries. Understanding how such constraints can alter the mitigating effects of vaccination is a crucial part of influenza preparedness plans. Mathematical models provide tools for government and medical officials to assess the impact of different vaccination strategies and plan accordingly. However, many existing models of vaccination employ several questionable assumptions, including a rate of vaccination *proportional *to the population at each point in time.

**Methods:**

We present a SIR-like model that explicitly takes into account vaccine supply and the *number *of vaccines administered per day and places data-informed limits on these parameters. We refer to this as the *non-proportional *model of vaccination and compare it to the proportional scheme typically found in the literature.

**Results:**

The proportional and non-proportional models behave similarly for a few different vaccination scenarios. However, there are parameter regimes involving the vaccination campaign duration and daily supply limit for which the non-proportional model predicts smaller epidemics that peak later, but may last longer, than those of the proportional model. We also use the non-proportional model to predict the mitigating effects of variably timed vaccination campaigns for different levels of vaccination coverage, using specific constraints on daily administration capacity.

**Conclusions:**

The non-proportional model of vaccination is a theoretical improvement that provides more accurate predictions of the mitigating effects of vaccination on influenza outbreaks than the proportional model. In addition, parameters such as vaccine supply and daily administration limit can be easily adjusted to simulate conditions in developed and developing nations with a wide variety of financial and medical resources. Finally, the model can be used by government and medical officials to create customized pandemic preparedness plans based on the supply and administration constraints of specific communities.

## Background

Influenza viruses continue to be a major cause of hospitalizations and deaths worldwide due to annual seasonal epidemics [1-6] and less-frequently occurring, but potentially more severe, pandemics [3,4,7-9]. Vaccination is one of the best tools health professionals have to prevent or mitigate influenza outbreaks [9,10]. Each year, vaccines are developed based on predictions about which influenza strains are likely to be circulating [9,11], and distributed prior to and during the influenza season. When there is good correspondence between the vaccine strains and the circulating strains, vaccination programs are highly effective in decreasing influenza-related hospitalizations and deaths, particularly in high-risk groups such as the elderly and children [12-14]. Vaccination prior to the first outbreak of a pandemic is usually not possible, due both to the unexpected nature of these outbreaks and the novelty of the viral strain responsible [7-9]. However, many influenza pandemics are characterized by multiple waves separated by months or years, which can allow time for vaccination development and administration in an attempt to mitigate subsequent outbreaks [15-19]. Unfortunately, resources are limited and many countries already struggle with insufficient doses of vaccines, as well as shortages in medical supplies, facilities, and workers to administer vaccines [20-22]. Resource limitations are likely to be even greater, and more widespread, in the case of a pandemic [8,20,22-24]. Even in the case of adequate resources, administration capacity is still limited; medical staff and facilities can only give a maximum number of vaccines per day [25-30], and that number may be small relative to the size of the population under consideration.

Understanding how vaccination resources can affect the size and dynamics of influenza outbreaks is crucial to outbreak preparedness [9]. Yet, many modeling studies examining the effectiveness of vaccination in mitigating outbreaks are based on questionable assumptions about supply and administration of vaccines. First, the assumed vaccine stockpiles are often very large relative to the size, and sometimes the location, of the considered population [31-34]. Second, previous studies modeling vaccination during a pandemic assume that vaccines are administered only to susceptible individuals [33,35-40]. This is problematic for several reasons. For one, medical professionals are rarely able to determine an individual's epidemiological status (i.e. susceptible, infected, recovered) prior to vaccination. Laboratory testing of individuals seeking vaccination is not required, nor recommended, for general use or clinical decision-making [41]. Also, individuals may not be aware of their own epidemiological status, either because they are asymptomatic or are unsure that an illness they recently experienced was due to the virus in question. Therefore, the only individuals who are likely not to seek or receive vaccination are those who are infected and symptomatic, and it is possible that many individuals with existing immunity get vaccinated. Considering only the susceptible population may underestimate the overall number of vaccines used, including those that are potentially wasted, during a vaccination campaign [42]. Finally, the administration of vaccines is usually modeled by specifying that a *proportion *of the population is vaccinated per day [31-40,43-48]. Such proportions may represent a very small or large number in comparison to the number of vaccines that can reasonably be administered on a daily basis. Although fixed-rate vaccination models exist (e.g. [49]), the formulations of which we are aware are not based on daily administration constraints. In practice, vaccination clinics are planned and run based on the *number *of people that can be vaccinated per day, given the availability and capacity of equipped facilities and medical professionals [25-30]. The concern is that modeling based on the assumptions discussed above may lead to innaccurate conclusions about the effects of vaccination programs on the size or progression of influenza outbreaks.

We developed an epidemiological model to investigate the spread of influenza that incorporates realistic constraints on vaccine supply and administration capacity. Our model allows the simulation of specific limits on the total number of vaccines available, the number that can be administered per day to a single population, the relative supply to different epidemiological classes, and the effects of the timing and duration of vaccination campaigns. Wherever possible, the values of these parameters were based on real and simulated data (see Table 1) from sources such as the Centers for Disease Control and Prevention (CDC), the World Health Organization (WHO), the Macroepidemiology of Influenza Vaccination (MIV)

**Table 1:** Parameters used in simulations

Parameter	Description	Value/Range	Source
*p*	probability of being confirmed	0.2 or 0.65	low probability [84,86], high probability [55]

*α*	relative infectiousness of unconfirmed class	0.5	based on reduced viral shedding [55-57]

*t_a_*	start of vaccination campaign (day)	20, 50, or 80	set to occur 10, 40, or 70 days after *t*_0_

*t_b_*	end of vaccination campaign (day)	Variable	depends on campaign start and duration

*t_d_*	depletion of vaccine stockpile (day)	Variable	depends on stockpile size; see

*t*_0_	time of initiating pulse (day)	10	arbitrary

*x*_0_	Amplitude of initiating pulse (individuals)	1	previous epidemiological model [62]

*a*	width of initiating pulse (days)	1	previous epidemiological model [62]

*b*	mean probability of infection per contact	0.476 or 0.346	adjusted as function of *p *so *R*_0_* = 2.0; see sea-sonal/pandemic *R*_0 _values [1,87]

*c*	rate of recovery (1/days)	1/7	based on symptoms, viral shedding, cytokine levels [55,58,59]

*N*	total population size	10^8^	e.g. Mexico, Phillipines [69,70]

	vaccine stockpile size	30 _* _10^6^	based on 30% coverage; see vaccine production/distribution data [21,60,61]

	maximum number of vaccines per day	10^5 ^- 10^7^	based on vaccination clinic modeling and clinic data [25-30]

*k*	proportion of eligible vaccinated per day	0.001 - 0.1 (0.1-10%)	see models using proportions in this range [17,36,38,44]

*δ*	infection-related death rate (1/days)	10^-6^	based on U.S. viral surveillance data [88]

*R_0_: Basic reproduction number, defined as the number of secondary infections occurring due to introduction of 1 infected individual into a susceptible population (for review see [89])

Study Group, and a variety of international researchers studying vaccine supply and administration. The vaccination parameters in our model can also easily be adjusted by public health officials or communities looking to examine the mitigating effects of vaccination given their specific supply and administration constraints.

We refer to our model as the *non-proportional *model of vaccination, since the administration of vaccines is limited by a daily maximum number rather than a proportion of the population, and compare the results to those obtained when vaccination is implemented using a proportional scheme. The results show that though the proportional and non-proportional models predict similar epidemics for a few different vaccination scenarios, there are regimes under which important differences in the dynamics of the two models are observed. Specifically, given particular combinations of vaccination campaign duration and daily supply limit, the proportional model predicts epidemics with larger final sizes and earlier peak times than those predicted by the non-proportional model. In addition, the proportional model predicts epidemics that last days less than in the non-proportional model. We argue that the non-proportional model provides more accurate information about the vaccine stockpiles and human resources needed to deal with real influenza outbreaks. Furthermore, our model can be used by government and medical officials to create customized pandemic preparedness plans based on the resources available to their specific communities.

## Methods

### Assumptions of the model

We developed a SIR-like epidemiological model to study the spread of influenza [50] (for a review of these models see [51]). The model describes the dynamics of susceptible (*S*), infected (*I*), recovered (*R*), vaccinated (*V*), and deceased due to infection (*D*) populations. In turn, the infected individuals are divided into two groups, infected confirmed (*I_C_*) and infected unconfirmed (*I_U_*). The confirmed group represents those individuals who test positive for influenza in a medical facility, while the unconfirmed group includes either asymptomatic individuals, or those whose symptoms are not severe enough to seek treatment and therefore never get tested.

Infections are assumed to be caused by an outbreak of a single influenza viral strain (e.g. pandemic H1N1 of 2009 [52-54]). A small number of individuals become infected at a time *t *= *t*_0_, referred to herein as the initial outbreak (e.g. the first H1N1 cases in the town of La Gloria in Veracruz, Mexico). For simplicity, all individuals in *S *are assumed to have an equal susceptibility to infection; age-related susceptibility and prior immune history are not considered. Individuals become infected through homogeneous mixing, at a rate proportional to the number of contacts between infected and susceptibles. Since several studies have shown a relationship between the degree of symptoms and the extent of viral shedding [55-57], we included a parameter, *α*, to simulate potentially reduced infectiousness of those in population *I_U_*. For most simulations *α *= 0.5, but using different values of *α *did not significantly change the results (data not shown). The majority of infected individuals recover at a rate *c*, which corresponds to a recovery period of 7 days based on data describing the progression of symptoms, viral shedding, and cytokine levels in influenza challenge studies [55,58,59]. Once recovered, individuals are assumed to have complete immunity against the virus that does not wane with time. Complete immunity, along with the fact that births are not included in the model, means that there is no continuing supply of susceptibles. Those who recover and were confirmed are represented by the population *R_C_*, while those who recover but were unconfirmed are represented by *R_U_*. A small number of people do not recover, however, and instead die as a result of infection, at a rate δ. The occurrence of deaths in the unconfirmed population is not meant to indicate that individuals may die without ever showing symptoms, but rather that some individuals may die before seeking medical attention and officially being classified as infected with influenza. This is particularly applicable to developing countries where many people may not have access to timely medical care. The rate of disease-related death, δ, is set low in our simulations to reflect a negligible death rate, but this parameter can be adjusted to simulate epidemics with higher mortality rates. For quantification purposes, a threshold of *n *individuals serves to find the start and end times of an epidemic. For the simulations presented herein, *n *= 10^4 ^(0.01% of the total population of 10^8 ^people).

### Assumptions about vaccination

In addition to acquiring immunity to the virus through infection, individuals can gain protection through vaccination. As previously explained, it is unrealistic to assume that only susceptibles will seek and receive vaccination during an epidemic. Therefore, in our model vaccines are distributed to the populations *S*, *I_U_*, and *R_C_*. Individuals from *I_U _*and *R_C _*are meant to represent those who become infected, but seek vaccination either because they are (were) unaware of their illness due to a lack of symptoms, or because the specific viral strain causing previously-experienced symptoms was not identified. As in recovered populations, vaccinated individuals are assumed to have total protection against the virus that does not wane. Vaccines that go to individuals in populations *I_U _*and *R_C _*are considered wasted, since immunity was already acquired through infection. We use the variable *V_U _*to keep track of vaccinated individuals from *I_U_*, and *VSC *to track those vaccinated from populations *S *and *IC*. Importantly, those vaccinated while belonging to the population *I_U _*continue to be infectious, and recover or die at the same rates as those who did not receive the vaccine.

The vaccine stockpile, , is limited. For most of the simulations shown here, it is assumed that  corresponds to a maximal coverage of 30% of the total population, a level of supply supported by vaccine production and distribution data for industrialized nations such as the U.S. [21,60,61]. The administration of vaccines starts at some time *t *= *t_a _*and effectively ends in one of two ways: (1) the vaccination campaign ends after some prescribed duration at time *t_b_*, or (2) the stockpile is depleted at time *t_d_*.

### Model

The dynamics of the model are defined by a system of non-autonomous ordinary differential equations of the form(1)(2)(3)(4)(5)(6)(7)(8)

so the infected, recovered, and vaccinated populations are given by(9)

The new infections per unit time are(10)

where *p *is the probability of being infected and confirmed, *c *is the rate of recovery of infected individuals (1/recovery time), *δ *is the infection-related death rate, *b *represents the mean probability of infection per contact. The function *δ*(*t*) defines a small bell-shaped pulse of the form(11)

that allows the insertion of (possibly more than one) infected individuals into an otherwise susceptible population at a time *t*_0_. The parameter *x*_0 _can be thought of as the number of infected individuals in the initial outbreak. The parameter *a *allows control over the time-duration of this initial outbreak. The use of this function adds the convenience of varying the starting point of the epidemic without assuming there are infected individuals at time *t *= 0, thus providing an anchor time around which vaccination, or other events, can be measured independently of the initial conditions of the system. This property is particularly useful to estimate the starting time of outbreaks which precede large epidemics (e.g. outbreaks in Mexico that preceded the H1N1 pandemic of 2009 [62]), or to simulate vaccination campaigns starting in anticipation of outbreaks (e.g. seasonal influenza or subsequent pandemic waves). Similar perturbation techniques are commonly used in computational physiology to simulate stimulation of excitable cells [63,64]. To ensure that use of the pulse does not result in negativity, we inserted a condition in the simulations to set *ϕ*(*t*) = 0 whenever . This control prevents more individuals from being removed from population *S *due to the pulse than are present.

The functions *v_S_*(*t*), *v_U_*(*t*), and *v_R_*(*t*) represent, respectively, expressions for the vaccines given each day to individuals from the *S*, *I_U_*, and *R_C _*populations, and will be defined in detail in the following section. The total number of vaccines administered *per day *is then(12)

Two extra (redundant) variables, *W *and *F*, are introduced in the simulations to quantify, respectively, the wasted vaccines (those given to *I_U _*and *R_C_*) and the cumulative number of infected individuals (the sum of all new infections) as a function of time:(13)(14)

### Proportional and non-proportional vaccination

The strategy adopted in most existing models of vaccination is to choose some constant, *k*, such that the number of people vaccinated per day is *kx*, where *x *is the vaccinable population [31-40,43,45-48]. As discussed earlier, many of these models also assume that vaccines are only administered to susceptible individuals, thereby setting the number of vaccinated people per day equal to *kS*. We refer to this scheme as the *proportional *model of vaccination. However, such a proportion may represent a very small or large number of people compared to the number of overall vaccines available and the number that can reasonably be administered in one day. Instead, we propose modeling vaccination by placing a limit on the number of daily vaccines before they are distributed between the epidemiological populations. We refer to the scheme presented herein as the *non-proportional *model of vaccination because the administration of vaccines depends on a daily limit, rather than on a proportion of the population.

#### Non-proportional vaccination rates

At each time step during the simulations, it is checked that there were enough vaccines in stock . If this condition is satisfied, the maximum number of vaccines per day, , is split according to the relative sizes of the different populations eligible for vaccination. To do so, we define the weights(15)

where *M*(*t*) = *S*(*t*) + *I_U_*(*t*) + *R_C_*(*t*) is the total number of people eligible for vaccination at time *t*. *M *is thus a decreasing function of *t *satisfying *M*(*t*) ≤ *N*. The maximum number of vaccines per day that each population can receive is(16)

Note that if either *S*, *I_U_*, or *R_C _*are zero, their corresponding weight would also be 0, and the maximum number of vaccines allocated for that population would be 0 as well.

Depending on the starting day of vaccination, *t_a_*, and on the maximum number of vaccines, , it is possible that some days there will be fewer individuals in a given epidemiological population than , the maximum number of vaccines available for that population. In other words, the vaccinable population could become negative if for *x *∈ {*S*, *I_U_*, *R*_C_},. To ensure that *x *never becomes negative due to the removal of vaccinated individuals, we assess whether each of these populations has enough people to be vaccinated at each step of the simulation. First, we calculate the vaccination-independent change in *x *at time *t*:(17)

We use *f *to estimate , the size of *x*(*t *+ *h*) where *h *is the maximum time step of the solver [65], and calculate the number of administered vaccines, *v_x_*(*t*), based on this estimate. To ensure the non-negativity of *x*(*t*), the number of vaccinated people removed from *x *cannot be larger than . In other words, the condition , must be satisfied so that *x*(*t*) ≥ 0 for all *t*. The number of vaccines administered per day to population *x *is then either: (i)  if , or (ii)  if . Note that according to condition (ii), if the estimated size of the population is zero, then there will be no vaccines given to that population. More formally, the number of vaccines administered to population *x *is defined as:(18)

Eq. (18) results in non-negative values of *x*(*t*) for all *t *for all *x *∈ {*S*, *I_U_*, *R_C_*} if the time step is small enough. As a rule of thumb, the maximum time step should be 10^-3 ^or smaller.

It follows from Eqs. (16) and (18) that  for all *t*. Note that saturation  cannot always be assumed because at some point there may not be enough individuals eligible for vaccination. This implies that there will be vaccines available for at least  days.

### Rationale of comparison between proportional and non-proportional vaccination

**Figure 1:**
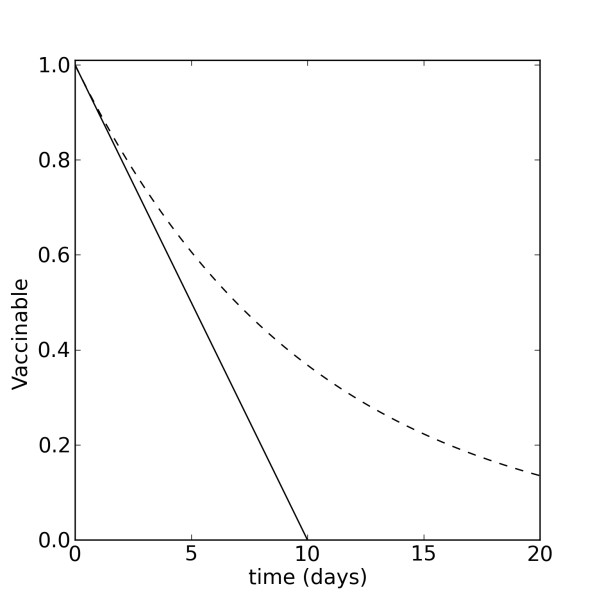
**Proportional and non-proportional decay of the vaccinable population**. Proportional decay (dashed line) is given by *x*(*t*) = *x*_0_*e^-kt^*, for *k *= 0.1. Non-proportional decay (solid line) is given by , where .

**Figure 2:**
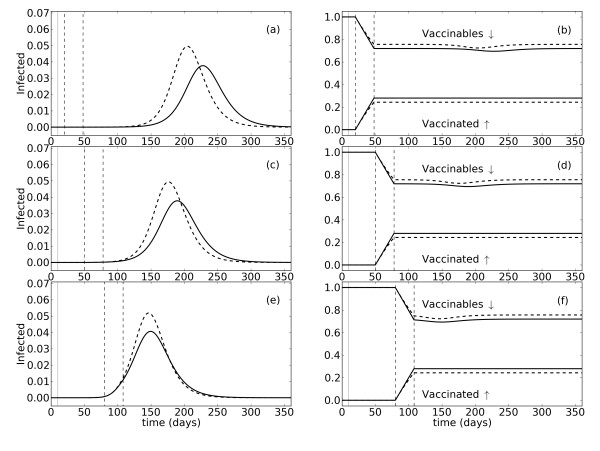
**Effects of vaccination in the proportional and non-proportional models for different campaign starts**. The proportional model is represented by dashed black lines and the non-proportional model by solid black lines. The graphs in the left column (**a, c, e**) show the proportion of infected people as a function of time. The graphs in the right column (**b, d, f**) show the proportion of the population vaccinated, and those still eligible for vaccination (vaccinable), over time. The initial population size is 108 people. Infected individuals are inserted into the susceptible population with a pulse on day 10 (*t*_0 _= 10; solid vertical gray line). The vaccination campaign is initiated on day 20 **(a, b)**, 50 **(c, d)**, or 80 **(e, f)**, and lasts 28 days. Start (*t_a_*)and stop (*t_b_*) times of the campaign are indicated by dashed vertical lines. Vaccination occurs at a rate of 1% of the eligible population per day (proportional; *k *= 0.01), or at a maximum of 10^6 ^vaccines per day (non-proportional, ).

For simplicity, consider an interval of time in which a population of size *x *contains no infected individuals, and assume all individuals are eligible for vaccination. If vaccines are supplied at the limit of capacity, the daily number of vaccines is a constant, namely, . In this case, the time course of *x *is described by a decreasing linear function of the form  (Figure 1, solid line; Figure 2, solid line in right column.) For comparison, let  where *x*_0 _is the initial size of the vaccinable population, and assume proportional vaccination . The decay in this case will be exponential at a rate *k *(Figure 1, dashed line; Figure 2, dashed line in right column). The difference in the time courses of these two decays, in particular the slower decay in the proportional model, may play an important role in shaping the mitigating effects of vaccination on a developing epidemic.

### Simulations

All numerical solutions of the model were obtained using Python 2.6 in a Lenovo T400 laptop with an Intel(R) Core(TM)2 Duo CPU T9600 at 2.8 GHz running Linux Kubuntu version 11. Simulations were performed using the solver *odeint *contained in the Python module scipy.integrate [66], which uses lsoda from the Fortran library odepack. The solver uses Adams method for nonstiff problems, and a method based on backward differentiation formulas for stiff problems. In addition, *odeint *allows time-step control to test numerical schemes and precision (simulations not shown). Figures were produced with the Python module matplotlib [67].

## Results

### Mitigating effects of vaccination in the proportional and non-proportional models

Initially, to compare the proportional and non-proportional models of vaccination, we conducted simulations in which the vaccination campaign lasted 30 days. This duration can be thought of as defined by constraints in medical personnel and other factors affecting the administration of vaccines (eg. pharmaceutical company distribution schedules, operation of health care facilities, governmental budgets, etc.). Assuming a stockpile equivalent to 30% of the total population, the distribution rate in the proportional model is set to 1% of the vaccinable population per day (*k *= 0.01). Equivalently, the maximum daily administration in the non-proportional model is then 10^6 ^vaccines . Figure 2 shows the infected, vaccinated, and vaccinables as functions of time for vaccination campaigns starting at three different times relative to the initial outbreak (*t*_0 _= 10): (1) 10 days after *t*_0 _(*t_a _*= 20), so that the entire campaign occurs well before the epidemic starts (Figure 2a, b), (2) 40 days after *t*_0 _(*t_a _*= 50), so the campaign ends shortly before the start of the epidemic (Figure 2c, d); and (3) 70 days after *t*_0 _(*t_a _*= 80), in which case the campaign is ongoing as the epidemic begins (Figure 2e, f). These campaign start times correspond to real-world scenarios. Early vaccination, as in campaign (1), is possible if a vaccine is already available and outbreaks are anticipated, as might be the case with seasonal influenza or with additional epidemic waves caused by a previously identified viral strain. Vaccination following detection of an outbreak, but before epidemic levels are reached (2), is again possible if a vaccine is available and efficient disease surveillance systems are present. However, if the surveillance system is not able to identify small outbreaks, or a vaccine is not immediately available, vaccination may not be implemented until after the epidemic starts (3).

The dynamics shown in Figure 2 reveal a number of similarities and differences between the proportional and non-proportional models of vaccination, which in turn depend on interactions between the start time and duration of the vaccination campaign, and the limitations on daily administration. During the early stages of vaccination, the rate of increase in the proportion of vaccinated individuals in the population is equivalent in the two models, as is the rate of decrease in the proportion of vaccinable people (Figure 2b, d, f). As time proceeds, however, the rate of vaccination in the proportional model slows down, while the rate in the non-proportional model holds fairly steady. Due to the duration of the campaign and the daily rate of administration, the campaign ends before the stockpile is depleted, and the proportional model does not have time to 'catch up' to the non-proportional model. Therefore, by the end of the campaign, a larger proportion of the population has been vaccinated in the non-proportional model. This increased vaccination coverage causes the epidemic in the non-proportional model to develop more slowly, increasing its duration, but ultimately resulting in a smaller and later peak in comparison to the proportional model (Figure 2a, c, e). The differences between the two models, with respect to the time course and severity of the epidemics, are largest when vaccination begins around the time of the initial outbreak (*t*_0_) and ends well before the epidemic hits (Figure 2a). As described previously, these differences are directly attributable to the increased number of vaccines administered in the non-proportional, relative to the proportional, model (Figure 2b). If vaccination occurs early, then the majority of those vaccinated are from population *S*, and therefore an increased level of coverage in the non-proportional model has a measurable effect on the epidemic development and size. Instead, if vaccination begins 40 days after *t*_0 _and the campaign ends shortly before the epidemic starts, the differences between the epidemics produced by the two models, especially with respect to the time course, are smaller (Figure 2c). Again, the proportion of people vaccinated in the non-proportional model is larger than in the proportional (Figure 2d). However, since vaccination begins some time after the initial introduction of infected individuals into the susceptible population, some of the additional vaccinated individuals in the non-proportional model are from populations *I_U _*and *R_C_*. In other words, the increased coverage in the non-proportional model has less of a mitigating effect when vaccination starts later because fewer of the vaccinated people are susceptibles. Finally, if vaccination begins 70 days after *t*_0 _and is ongoing as the epidemic starts, the number of vaccinated individuals from populations *I_U _*and *R_C _*is now even greater, essentially diluting the effect of the increased coverage in the non-proportional model (Figure 2f) further. Therefore, the models produce epidemics which are very similar in their time course, and differ primarily in their peak size (Figure 2e).

We also compared the proportional and non-proportional models when the same total number of vaccines was administered in each. This was accomplished by setting the campaign duration and daily administration limits such that all the vaccines in the stockpile were used. In this way, the same level of coverage is achieved, though the proportional model always takes longer to reach this level due to slowing of the administration rate over time. As long as vaccination occurs early, before the number of infections increases substantially, the additional time needed for the proportional model to administer the whole stockpile has a minimal effect on the final coverage in the susceptible population. Thus, the epidemics produced by the two models are nearly identical (Additional File 1). However, if vaccination does not begin until after the epidemic is underway, the increasing number of infections over time starts to affect the level of coverage achieved in *S*. The slower rate of administration in the proportional model results in an increased number of susceptibles at certain times during the epidemic, relative to the non-proportional model. This increased susceptibility results in a slightly faster epidemic development than is seen in the non-proportional model (Additional File 1e), and occurs despite the fact that the final number of vaccines administered is the same for both models.

Additional simulations revealed similar results for several combinations of campaign duration and daily administration limit (see subsequent section for more details). In general, if the campaign ends before the vaccine stockpile is depleted, more vaccines are always administered in the non-proportional model, and the epidemics produced by the two models are different with respect to timing and severity. Instead, if the same total number of vaccines is administered in each model, the epidemics do not differ unless vaccination begins very late.

### Comparison of the models for variable daily administration and campaign duration

To further explore in both models the effects of changing the timing of the vaccination campaign, we systematically varied *t_a _*under three different scenarios of daily administration capacity and campaign duration. These three scenarios can be thought of as ranging from 'conservative' to 'aggressive' in terms of the number of vaccines administered within a given time period, and are as follows: (1) a 56 day campaign vaccinating 0.1% (proportional) or a maximum of 10^5 ^vaccines (non-proportional) per day; (2) a 28 day campaign vaccinating 1% or a maximum of 10^6 ^vaccines per day; (3) a 3 day campaign vaccinating 10% or a maximum of 10^7 ^vaccines per day. For each of these combinations of campaign duration and daily administration limit, the non-proportional model administers a larger total number of vaccines than the proportional model. The data obtained from these simulations were used to calculate the final size, peak size, time to peak, and epidemic duration as a function of the difference between *t_a _*and *t*_0_.

**Figure 3:**
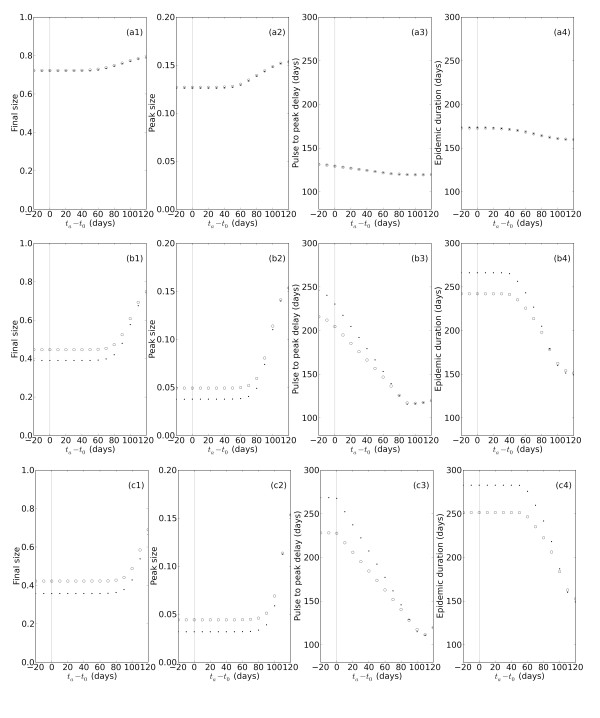
**Effects of vaccination in the two models for different administration rates and campaign durations**. Epidemic measures are shown for proportional (open circles) and non-proportional (filled dots) models. Final size, peak size, peak time, and epidemic duration are plotted as a function of the difference between the vaccination start time (*t_a_*) and the onset of the initial outbreak (*t*_0_; solid gray line). The vaccination campaign durations and daily administration rates are as follows: (1) 56 day campaign with k = 0.001 (proportional) or  (non-proportional) **(a1-a4)**, (2) 28 day campaign with k = 0.01 or **(b1-b4)**, and (3) 3 day campaign with k = 0.1 or **(c1-c4)**.

The conservative campaign (1) results in no detectable differences between the proportional and non-proportional models on any quantified measure (Figure 3a1-a4). Under this low level of daily administration, the decay in the vaccinable population is very slow in both models and there is not time for the decay rates to diverge before the campaign ends. The level of vaccination coverage in both models is therefore the same and in turn, the number of susceptible people that get infected over time is very similar. In addition, because so few people are vaccinated overall, the mitigating effects of vaccination are minimal and each measure varies little as a function of the timing of the vaccination campaign (*t_a _*- *t*_0_). Late vaccination start times caused the peak and final size to increase, and the peak time and epidemic duration to decrease, but only marginally.

Though the models behave similarly when examined under the conservative vaccination regime, the moderate regime (2), equivalent to the campaign used for the simulations in Figure 2, reveals important differences between the models on all four measures (Figure 3b1-b4). For early vaccination starts, final and peak sizes are smaller, while peak times and epidemic durations are larger, in the non-proportional than the proportional model. As discussed previously, these differences result from the higher level of vaccine coverage achieved in the non-proportional, relative to the proportional, model. With later vaccination starts, due to the increasing number of vaccinated individuals from populations *I_U _*and *R_C_*, the differences between the models decrease until the models converge on most measures. Interestingly, with respect to epidemic duration, the two models not only converge, but reverse their respective relationship; epidemic durations are slightly smaller in the non-proportional model for very late vaccination start times (Figure 3b4). For the aggressive campaign (3), the proportional and non-proportional models also differ with respect to the time course and severity of epidemics (Figure 3c1-c4). The differences are the same as those seen under the moderate vaccination regime, namely smaller final and peak sizes, and larger peak times and epidemic durations, in the non-proportional model. The same relationship is also observed between the magnitude of these differences and the timing of the vaccination campaign relative to the initial outbreak. Later vaccination start times result in a convergence of the proportional and non-proportional models on all measures.

We also performed these same simulations for different combinations of campaign duration and daily administration limit. The vaccination scenarios are as follows: (1) a 300 day campaign vaccinating 0.1% (proportional) or a maximum of 10^5 ^vaccines (non-proportional) per day; (2) a 35 day campaign vaccinating 1% or a maximum of 10^6 ^vaccines per day; (3) a 5 day campaign vaccinating 10% or a maximum of 10^7 ^vaccines per day. Under these conditions, the vaccine stockpile is depleted before the end of the campaign, resulting in the same number of vaccines being administered in each model. The epidemics produced by the two models are thus not measurably different, as previously explained, except for the latest vaccination start times under the moderate regime (Additional File 2).

### Effects of non-proportional vaccination for different levels of population coverage

**Figure 4:**
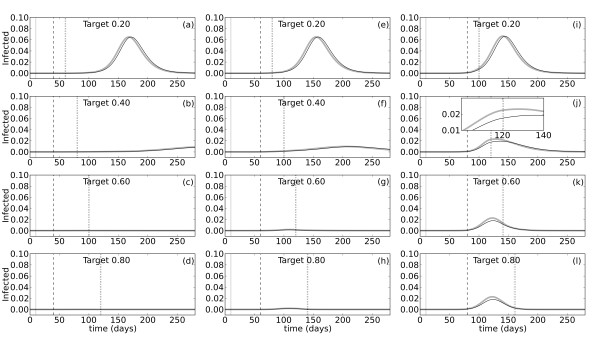
**Effects of vaccination in the non-proportional model given different levels of population coverage**. Simulations were performed using the non-proportional model of vaccination with . The pulse inserting infected individual(s) into the susceptible population occurs at *t*_0 _= 10 (gray solid vertical lines). The proportion of people infected over time is plotted for vaccination start times, *t_a _*= 20 **(a**-**d)**, *t_a _*= 50 **(e**-**h)**, and *t_a _*= 80 **(i**-**l)**. Start times are indicated with dashed lines in each panel. The target level of vaccination coverage in the total population varies between 20% and 80%, as indicated. Dotted lines mark the end of the vaccination campaign when the target coverage level is reached. The probability of being confirmed, *p*, is set at either 0.20 (thick gray lines) or 0.65 (thin black lines), and *b *adjusted accordingly such that *R*_0 _= 2.0 for all simulations. Note that the gray and black lines overlap. The inset in panel **(i) **illustrates the change in the epidemic dynamics at the time vaccination ends.

Finally, we investigated the mitigating effects of vaccination in the non-proportional model when different target levels of total population coverage are met at different times relative to the initial outbreak. All simulations shown previously assume a maximum of 30% population coverage due to the size of the stockpile . To simulate vaccination campaigns with higher levels of coverage, we increased  to a size equivalent to 20, 40, 60, or 80% of the population. In addition, since the effective coverage in the susceptible population could be altered if a large number of vaccines go to infected unconfirmed individuals, we performed the same simulations for *p *= 0.2 or *p *= 0.65 (the value used in all previous simulations). In other words, unconfirmed cases represent either 80% or 35% of total infections, respectively. The probability of infection per contact is adjusted so that the basic reproductive number (*R*_0_) is the same in both simulations. Figure 4 shows the proportion infected over time when 20% (a, e, i), 40% (b, f, j), 60% (c, g, k), or 80% (d, h, l) of the population is vaccinated with a maximum of 10^6 ^vaccines per day. The vaccination campaign starts 10 (Figure 4, left column), 40 (middle column), or 70 days (right column) after the initial oubreak at day *t*_0 _= 10. Vaccination ends when the target level of coverage is achieved i.e. the higher the coverage, the longer the campaign.

The results show that even if 20% of the population is vaccinated, there is still a sizeable epidemic (Figure 4a, e, i). Furthermore, starting vaccination earlier does not dramatically decrease the number of infections, though it does delay the start of the epidemic. At 40% coverage, the number of infections is highly dependent on when the vaccination campaign begins (Figure 4b, f, j). When vaccination starts only 10 days after the pulse, it successfully prevents an epidemic from occurring (Figure 4b). A start time of 30 days later does not prevent a small number of late-occurring infections, but is still a highly effective mitigation strategy (Figure 4f). A full-blown epidemic does occur if vaccination starts as late as 70 days after the initial outbreak, but the peak size is smaller than that observed with only 20% coverage (Figure 4j). At 60% coverage the results of vaccination are similar, though even more effective (Figure 4c, g, k). Starting vaccination 10 or 40 days after the pulse completely quells the outbreak (Figure 4c, g), while beginning 70 days later does not prevent a small, but short, epidemic from occurring (Figure 4k). Increasing coverage from 60% to 80% does not confer much additional benefit with respect to either arrival, size, or time course of the epidemic (Figure 4d, h, l), but does result in a larger number of wasted vaccines (Additional File 3). The results are similar for the two values of *p *for all simulations, regardless of the level of coverage or the vaccination start time. In other words, increasing the proportion of unconfirmed cases does not alter the mitigating effects of vaccination. Decreasing the value of *p*, and thereby increasing the number of vaccines going to population *I_U_*, increases the number of wasted vaccines only slightly (Additional File 3). Thus, for the parameter choices made here, the majority of wasted vaccines go to population *R_C_*, and changing the number going to *I_U _*does not significantly change the level of coverage in *S*.

## Discussion

Epidemiological models can be vital tools for government and medical professionals who need to understand the spread of diseases and the effects of mitigating strategies, such as vaccination, to form public policies that will help reduce the burden of disease [9,68]. These models are only useful, however, if they generate accurate predictions and the tools they provide can be applied to real situations. We argue that many epidemiological models examining the mitigating effects of vaccination incorporate a number of unrealistic assumptions. These include: (1) large vaccine stockpiles, (2) vaccination of only the susceptible class, and (3) the administration of vaccines based on a proportion of the population, without taking into account daily administration constraints. We constructed a model designed to incorporate more realistic assumptions about the supply and distribution of vaccines.

### Vaccine stockpile size

We placed a conservative, but reasonable, limit on the total number of vaccines available at 30 ×10^6^; enough to cover 30% of the population. A stockpile covering this percentage of the population is in agreement with seasonal influenza vaccine production and distribution for resource-rich countries, such as the U.S. [21,60,61]. From 2000 to 2009, the number of seasonal vaccines produced annually for use in the U.S. did not exceed 140.6 million (~46% of the total population of 307 million [69,70]), and in most years was far less at an average of 99.7 million (~32%) [60]. Even Canada, with the largest per capita annual influenza vaccine distribution of all countries surveyed, distributed only enough vaccines as of 2003 to cover ~34% of their population [21]. For many other countries in the world, annual vaccine supplies are nowhere near enough to cover 30% of their population, primarily due to the lack of vaccine production capabilities and the resources needed to buy vaccines from producing countries [20-22,24]. As of 2003, over 60% of the total influenza vaccines distributed annually went to countries comprising only 12% of the world population, leaving 88% of the world facing potential vaccine shortages [20,21].

In 2009, the emergence of a novel influenza A (H1N1) strain [52-54] tested the world's ability to rapidly develop and distribute large numbers of vaccines to combat an ongoing pandemic [71,72]. Though vaccine development was quick and successful, production levels fell far short of initital estimates [72], and even developed countries struggled to secure sufficient vaccines to cover their populations before subsequent waves hit [54,73,74]. By the time the fall wave peaked in the U.S. in mid October, only ~10 million vaccines were available [74]; Canada had distributed less than 6 million vaccines [75] when their second wave peaked just a few weeks later [73,76]. It wasn't until mid December 2009 that the number of vaccines available in the U.S. reached levels high enough to cover 30% of the population [74]. Canada had enough vaccines by the first week of December to cover a larger percentage of their population (~67%) [75], but were subsequently accused of wasting resources as most vaccines arrived too late to have an effect during the peak periods of influenza activity [73,77]. For developing nations the situation was much worse [54,62,72]. For example, the Mexican government indicated in June of 2009 that they would have 30 million H1N1 vaccines (enough to cover ~30% of the population) [62,78,79]. Yet, three waves of infection had already hit by the time the first 650 thousand vaccines arrived in late November [62]. By December 1, 2009 the global production of H1N1 vaccines had reached 534 million, enough to cover only ~8% of the world population [72]. Many developing countries did not have any vaccines until January 2010 [72]. The production and distribution of seasonal and pandemic vaccines show that stockpiles assumed in many models are in excess of what is usually available. Some studies modeling vaccination in simulated U.S. populations have considered upper limits of 300-400 million vaccines (100%+ coverage) [31,33]; levels more than double the U.S. annual production [60] and close to the *global *production of seasonal influenza vaccines [61,72,80]. Other models looking at vaccination during a pandemic have assumed population coverage levels as high as 70-90% [32,34,35,38,39,81], levels not achieved by many wealthy nations during the most recent pandemic, and currently out of reach for most developing nations [20-22,24].

In 2004, the WHO published two separate reports on guidelines for vaccine use during a pandemic [9] and pandemic preparedness in countries with limited resources [82]. In both reports, they remarked on the usefulness of mathematical models for examining different pandemic scenarios and the effectiveness of strategies, such as vaccination, in mitigating outbreaks [9,82]. However, they lamented that, "...no scenarios appropriate to developing countries are readily available" (see [9], pg. 5), a sentiment echoed by others [22]. In 2005, a model of a developing country in Southeast Asia, Thailand, was used to examine vaccination strategies in the context of an avian influenza epidemic [32]. To realistically model factors such as age and household-size distributions, and population mixing, they relied on census data from the Thai government and social network studies of Thai communities. Yet, they assumed that vaccination coverage in the population of 500 thousand people was 50-70%, equivalent to a total of 250-350 thousand vaccines [32]. Between 2000 and 2003, a total of 64-253 thousand vaccines were distributed to the entire WHO-defined region of Southeast Asia with a population of over 1.5 million [20,21]. Specifically in Thailand, between 1997 and 2003 the number of vaccines distributed per capita was ~1 per 1000 people (0.1% coverage) [20,21]. Thus, the crucial detail of setting a limit on the vaccine stockpile that was realistic for Thailand was neglected in the model.

Though many of the parameters in our model listed in Table 1 including vaccine stockpile, were set to values observed mostly for developed countries, the model is easily adjusted to apply to developing countries. The size of the stockpile, , can be set to correspond to very low levels of population coverage.

In these cases, we would expect to see that vaccination campaigns would have less of a mitigating effect, resulting in larger epidemics with earlier time to peak than the simulations shown here. However, it should be noted that the general results regarding the comparison between the proportional and non-proportional models still hold for a wide range of stockpile sizes.

The vaccine stockpile can also be increased to model the effects of increasing population coverage, as done for the simulations in Figure 4. As expected, we find that increasing the percentage of the population protected by vaccination decreases the size and duration, and delays the arrival, of the epidemic. At only 40% coverage the epidemic can be completely prevented, if vaccination begins early enough. Even vaccination beginning around the start of the epidemic can help significantly to control the number of infections. However, if vaccination cannot begin sooner than this time, it is important to note that increasing coverage from 60% to 80% in our simulations does not further decrease the size or duration of the epidemic. Therefore, continuing campaigns during an epidemic until 80% of the population is vaccinated may result in a large number of vaccines being wasted in return for little benefit (Additional File 3).

### Vaccination of multiple epidemiological populations

In our model, not only susceptible individuals, but also unconfirmed infected and confirmed recovered people, are eligible for vaccination. There is little documentation about the actual number of unconfirmed and recovered people who do get vaccinated. However, in the context of the 2009 H1N1 vaccination campaigns, the CDC recommended that those who had influenza-like symptoms (i.e. recovered) should still get vaccinated, if medically indicated, due to uncertainty about which specific viral strain caused the illness [83]. In addition, they stated that even in those cases when infection by 2009 H1N1 had been confirmed by laboratory testing, it was an individual's choice as to whether to receive vaccination. Thus, recovered people, even those who were previously symptomatic and confirmed, can seek and receive vaccination. In this respect, the eligible population in our model is reasonable.

Expanding vaccine administration to include the unconfirmed population, in particular, also allowed us to examine the role that a high rate of asymptomatic cases might play in the context of vaccination efforts [42]. This is particularly relevant for 2009 H1N1, which was reported by some as characterized by a large proportion of asymptomatic cases [84]. However, in our simulations, decreasing the probability of being confirmed, *p*, from 0.65 to 0.20 does not alter the mitigating effects of vaccination (Figure 4). The majority of wasted vaccines in our model go to the confirmed recovered population (Additional File 3) so that the number of vaccines going to the unconfirmed class does not significantly change the level of coverage in the susceptible population. Larger effects may be seen when the vaccine stockpile is severely limited, as could be the case for developing countries.

### Limited number of vaccines administered per day

The key observation prompting the development of the model presented here was that most existing models of vaccination of which we are aware distribute vaccines based on a proportion of the eligible population [31-40,43-48]. Considering that vaccination clinics operate with a finite number of medical professionals for a finite number of hours, however, it is clear that distribution happens in practice based on the number of vaccines that can be administered per day [25-30]. Pandemic prepareness plans devised by county health departments often calculate the necessary length of vaccination campaigns using a formula based on daily administration capacity (e.g. see [28]). Therefore, we model vaccination by placing a limit on the number of daily vaccines (non-proportional model).

Initially, to compare the proportional and non-proportional models of vaccination we assumed that the daily limit was 10^6 ^vaccines (a value corresponding to 1% of the population). A number of resources discuss the administration capabilites needed to distribute specific numbers of vaccines [25-30]. For example, a model by Aaby et al. [25] predicted that 316 people could be vaccinated per hour with the aid of 18 nurses. Thus, one clinic operating for 8 hours could vaccinate 2,528 people per day. To reach a daily administration of 10^6 ^vaccines would therefore require more than 395 clinics and more than 7,110 nurses (or other capable medical professionals). Similarly, estimates from the CDC are that a single vaccinator at a station can administer 30 vaccines per hour. With 16 hours of operation and 4-8 stations, one clinic could administer 1,900-5,000 vaccines per day. At this rate, it would require 200-526 clinics and 1,600-8,416 vaccinators to distribute 10^6 ^vaccines per day. Data from actual vaccination clinics reveal a similar story. In 2004, a mass vaccination clinic in Maryland administered vaccines to more than 3,000 people in a single day with a workforce of 36 nurses, and 38 additional staff. Thus, to reach 10^6 ^vaccines per day would have required more than 333 clinics, nearly 12,000 nurses, and over 12,654 additional workers. Based on all these estimates, if instead a daily rate of 10^7 ^vaccines (10% of the population) is desired, the clinics and staff needed would increase to the thousands and hundreds of thousands, respectively. For these reasons, we set 10^7 ^(10%) as the upper limit of daily administration.

In some nations, where the medical resources are sufficient and the infrastructure is present, high daily rates of vaccine administration may be possible. Even so, mass vaccination campaigns often require extensive planning and agency cooperation to carry out [27-29,74], and are not intended to be sustained for long periods of time. In addition, long campaigns at high rates of daily administration would require very large vaccine stockpiles. Therefore, we limited high daily administration (10^7 ^or 10%) campaigns to a small number of days (≤5). For many developing nations with minimal resources, however, daily vaccine administration capacity is much more limited [22]. To model the mitigating effects of vaccination in these nations the daily administration limit, , is easily lowered in the model to reflect the medical facilities, workforce, and other resources available.

### Comparison of models in context of total vaccines administered

We predicted, based on the solutions of the equations representing the proportional and non-proportional models, that the different decays in the vaccinable population (Figure 1), would lead to distinct epidemic dynamics. This prediction was confirmed under several vaccination scenarios in which the campaign duration and daily administration limit were such that vaccination ended before the stockpile was depleted (Figure 2 and Figure 3). Under such conditions, the non-proportional model always administers a larger total number of vaccines, which results in smaller and later, but sometimes longer, epidemics than in the proportional model. This is true for both moderate and high levels of daily vaccine administration, and is more pronounced the sooner vaccination starts after the initial outbreak.

If instead vaccination continues until the stockpile is depleted, the same total number of vaccines are administered in each model, and the epidemics produced are very similar in time course and severity (Additional Files 1, 2). The only differences between the two models in these cases are seen under the moderate regime when vaccination starts after the epidemic hits. Then, the slower rate of vaccine administration in the proportional model means that there is a small increase in the susceptiblity of the population during the epidemic, which results in a slightly faster and more severe epidemic than in the non-proportional model. In contrast, at low daily rates of administration, the models do not differ much on any measure, regardless of whether the same total number of vaccines in administered. This occurs because so few people are vaccinated per day that the proportional and non-proportional decays in the vaccinable population do not have time to diverge. Thus, the models achieve the same level of vaccine coverage even if the stockpile is not depleted.

### Difference in epidemic duration

One of the largest differences between the two models when different total numbers of vaccines are administered is the epidemic duration. This stems from the increased coverage of the population in the non-proportional model, which allows the epidemic to develop more slowly, but can also cause it to last tens of days longer than predicted by the proportional model. Interestingly, a similar effect was found in an agent-based model of influenza (for a review of these types of models see [85]). Hartvigsen et al. [81] showed that for certain schemes of connectivity between the agents (individuals), increasing vaccination coverage from 0% through ~30% increased the epidemic duration; an effect that occurred due to slowing of the epidemic development. Having the benefit of a smaller, delayed peak could therefore come at a cost, since it could result in a sustained burden on the healthcare system. Models that make more accurate predictions about the length of epidemics will allow health care professionals and medical facilities to prepare accordingly.

## Conclusions

We developed a model to explore the mitigating effects of vaccination on influenza outbreaks. We argue that our model constitutes a theoretical improvement over existing models, with advantages that include data-informed parameter choices, vaccination of multiple epidemiological classes, a reasonable vaccine stockpile, limits on the number of vaccines administered per day, and ways to estimate wasted resources. In particular, the non-proportional vaccine administration implemented in our model may provide more accurate predictions of the mitigating effects of vaccination than proportional models, particularly when moderate or high levels of daily administration are considered. In addition, supply and daily administration capacity can be adjusted to study vaccination strategies in developing nations with limited resources. Government and medical officials can also use the tools provided here to create influenza preparedness plans for specific communities based on their available resources.

## Competing interests

The authors declare that they have no competing interests.

## Authors' contributions

All authors contributed equally to designing the study, writing the code, performing the simulations, and writing the paper. All authors read and approved the final manuscript.

## Pre-publication history

The pre-publication history for this paper can be accessed here:

http://www.biomedcentral.com/1471-2334/11/207/prepub

## Supplementary Material

Additional file 1**Comparison of the proportional and non-proportional models when the stockpile is depleted**. The proportional model is represented by dashed black lines and the non-proportional model by solid black lines. The graphs in the left column (**a, c, e**) show the proportion of infected people as a function of time. The graphs in the right column (**b, d, f**) show the proportion of the population vaccinated, and those still eligible for vaccination (vaccinable), over time. The initial population size is 10^8 ^people. Infected individuals are inserted into the susceptible population with a pulse on day 10 (*t*_0 _= 10; solid vertical gray line). The vaccination campaign is initiated on day 20 **(a, b)**, 50 **(c, d)**, or 80 **(e, f)**, and lasts 40 days such that all the vaccines are used in both models. Start (*t_a_*)and stop (*t_b_*) times of the campaign are indicated by dashed vertical lines. Vaccination occurs at a rate of 1% of the eligible population per day (proportional; *k *= 0.01), or at a maximum of 10^6 ^vaccines per day (non-proportional, ).Click here for file

Additional file 2**Effects of vaccination for administration rates and campaign durations resulting in stockpile depletion**. Epidemic measures are shown for proportional (open circles) and non-proportional (filled dots) models. Final size, peak size, peak time, and epidemic duration are plotted as a function of the difference between the vaccination start time (*t_a_*) and the onset of the initial outbreak (*t*_0_; solid gray line). The vaccination campaign durations and daily administration rates are as follows: (1) 300 day campaign with k = 0.001 (proportional) or  (non-proportional) **(a1-a4)**, (2) 40 day campaign with k = 0.01 or **(b1-b4)**, and (3) 5 day campaign with k = 0.1 or **(c1-c4)**.Click here for file

Additional file 3**Wasted vaccines for different population coverage levels, vaccination start times, and proportion of unconfirmed cases**. Simulations were performed using the non-proportional model of vaccination with . The pulse inserting infected individual(s) into the susceptible population occurs at *t*_0 _= 10 (gray solid vertical lines). The proportion of vaccines wasted over time is plotted for vaccination start times, *t_a _*= 20 **(a**-**d)**, *t_a _*= 50 **(e**-**h)**, and *t_a _*= 80 **(i**-**l)**. Start times are indicated with dashed lines in each panel. The target level of vaccination coverage in the total population varies between 20% and 80%, as indicated. Dotted lines mark the end of the vaccination campaign when the target coverage level is reached. The probability of being confirmed, *p*, is set at either 0.20 (thick gray lines) or 0.65 (thin black lines), and *b *adjusted accordingly such that *R*_0 _= 2.0 for all simulations.Click here for file
