# Supplementary figures and images for: Mitigating effects of vaccination on influenza outbreaks given constraints in stockpile size and daily administration capacity
Source: BMC Infect Dis. 2011 Aug 1;11:207. doi: 10.1186/1471-2334-11-207 (PMC3162903; doi:10.1186/1471-2334-11-207)

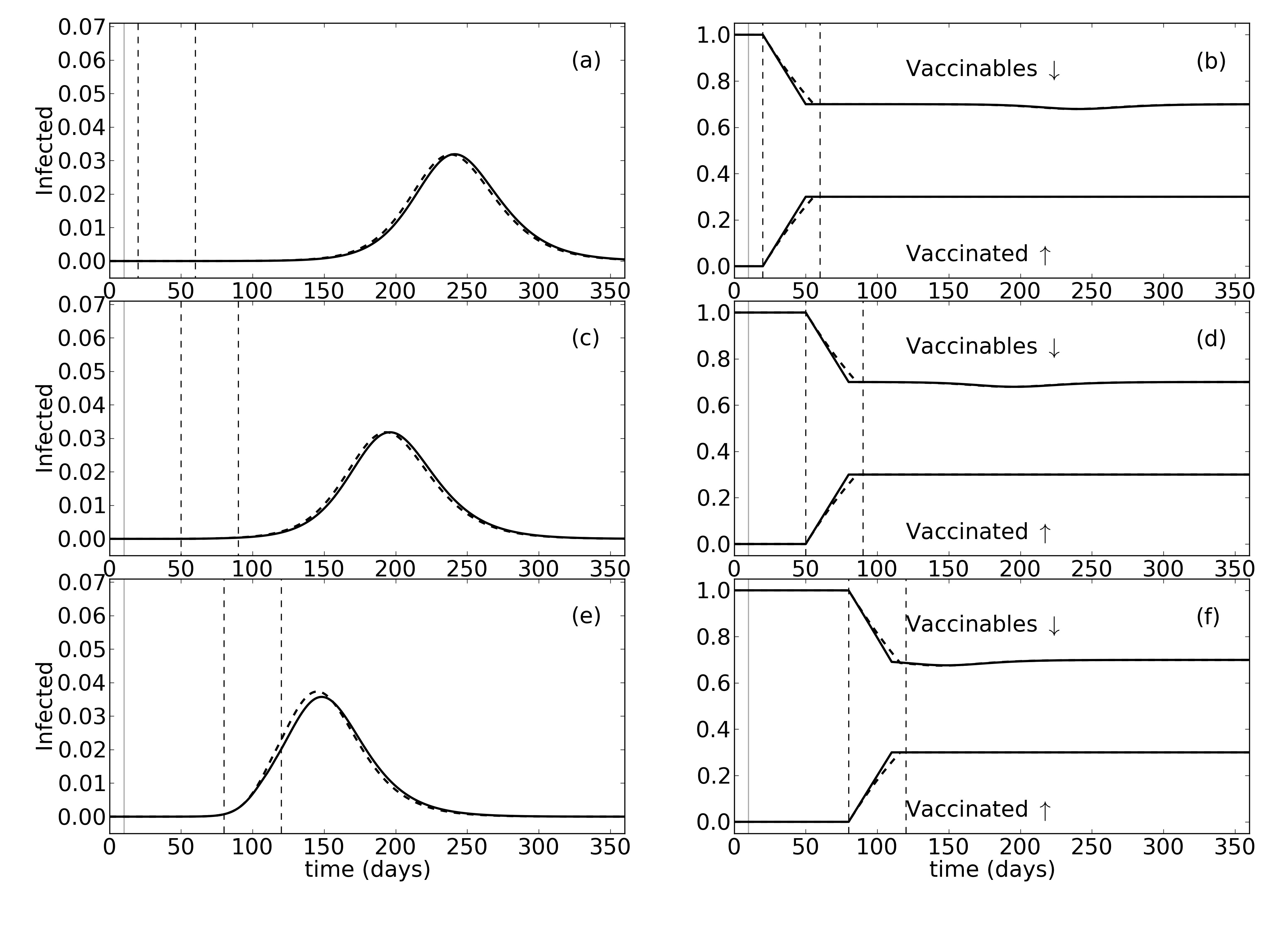

Supplement: Additional file 1 — Comparison of the proportional and non-proportional models when the stockpile is depleted. The proportional model is represented by dashed black lines and the non-proportional model by solid black lines. The graphs in the left column (a, c, e) show the proportion of infected people as a function of time. The graphs in the right column (b, d, f) show the proportion of the population vaccinated, and those still eligible for vaccination (vaccinable), over time. The initial population size is 108 people. Infected individuals are inserted into the susceptible population with a pulse on day 10 (t0 = 10; solid vertical gray line). The vaccination campaign is initiated on day 20 (a, b), 50 (c, d), or 80 (e, f), and lasts 40 days such that all the vaccines are used in both models. Start (ta)and stop (tb) times of the campaign are indicated by dashed vertical lines. Vaccination occurs at a rate of 1% of the eligible population per day (proportional; k = 0.01), or at a maximum of 106 vaccines per day (non-proportional, ). [file 1471-2334-11-207-S1.PNG]

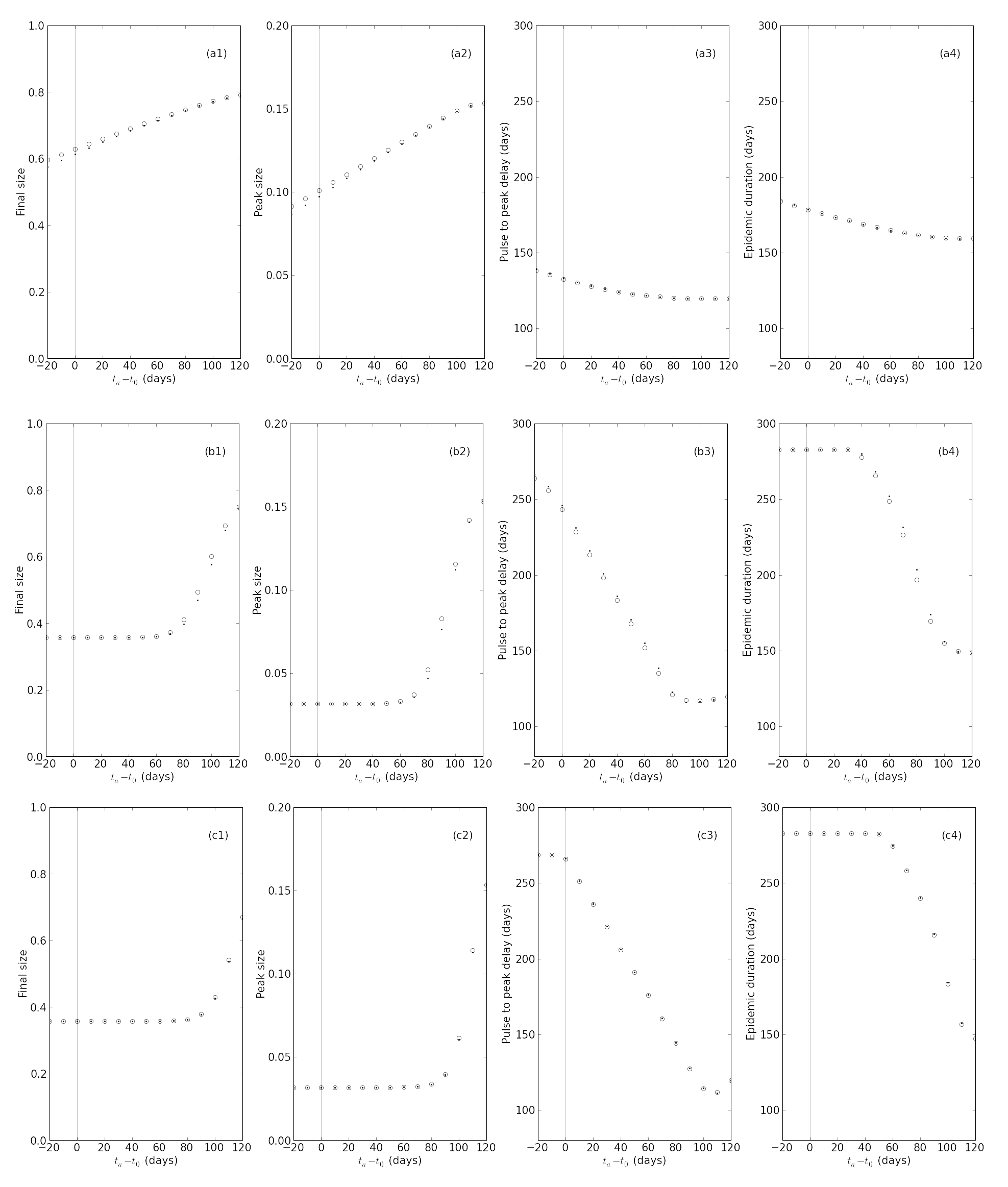

Supplement: Additional file 2 — Effects of vaccination for administration rates and campaign durations resulting in stockpile depletion. Epidemic measures are shown for proportional (open circles) and non-proportional (filled dots) models. Final size, peak size, peak time, and epidemic duration are plotted as a function of the difference between the vaccination start time (ta) and the onset of the initial outbreak (t0; solid gray line). The vaccination campaign durations and daily administration rates are as follows: (1) 300 day campaign with k = 0.001 (proportional) or (non-proportional) (a1-a4), (2) 40 day campaign with k = 0.01 or (b1-b4), and (3) 5 day campaign with k = 0.1 or (c1-c4). [file 1471-2334-11-207-S2.PNG]
